# The Use of Internet and Social Media for Health Information and Its Consequences Among the Population in Saudi Arabia

**DOI:** 10.7759/cureus.18338

**Published:** 2021-09-27

**Authors:** Sarah A AlMuammar, Afnan S Noorsaeed, Raghad A Alafif, Yumna F Kamal, Ghaida M Daghistani

**Affiliations:** 1 Family Medicine, King Abdulaziz University, Jeddah, SAU; 2 Medicine and Surgery, King Abdulaziz University Faculty of Medicine, Jeddah, SAU

**Keywords:** social media platform, internet, health information, whatsapp, web search, telemedicine

## Abstract

Background: The Internet is being increasingly used in our lives. Along with Internet use, social media sites are especially popular and are used by many people on a daily basis.^ ^Many studies were conducted to see the people’s perception and their attitude towards the use of the Internet for health information. Such studies were also carried out in Saudi Arabia, but most have been limited to a specific target sample of the population. Our study aimed to assess the perception towards and use of different platforms and search engines to seek health information in Saudi Arabia.

Method: A cross‐sectional study was conducted in Saudi Arabia during the year 2021. An Arabic online questionnaire using Google forms was sent to a randomized sample. Microsoft Excel 2016 was used for data entry, and statistical analysis was performed using IBM© SPSS© Statistics version 25 (IBM© Corp., Armonk, NY, USA).

Result: Among the 1363 participants, 56.2% were females and 43.8% were males with a mean age of 30.73 ± 12.3. The majority were living in Western region. The most used social media platforms were WhatsApp (91.5%), YouTube (84.6%), and Twitter (82.6%), respectively. The most common medical websites browsed were the Saudi Ministry of Health (67%) and the Food and Drug Administration (54.4%). Some 40.1% of the participants had a medical consultation online from a doctor, and most of them (67.8%) trusted the online physician. Finally, most of the participants (90.9%) thought that health information on the Internet or social media contributes to raising the level of health awareness among the general population. There was a significant relationship between educational level and gender and online health-seeking behavior (p > 0.05).

Conclusion: The study reinforced that health information that is sought from the Internet and social media platforms has a great impact on the population, emphasizing the need for credible information sources and how to access them.

## Introduction

The Internet is being increasingly used in our lives; it is a global network that enables us to obtain different types of information, from topics such as education and entertainment [1-2]. Along with Internet use, social media sites such as WhatsApp, Twitter, Instagram, and Snapchat are especially popular and are used by many people on a daily basis [3]. In developed countries, three in four citizens are online and approximately one in four people are online in the world [4].

According to International Telecommunication Union (ITU) between 56% and 79% of users get health information online in the United States [5]. An earlier study found that 47% of the South Asian population in Canada seek the Internet for obtaining health information [6]. Many users seek these platforms to reach for health information related to their condition or for others or just out of curiosity [7-8]. With an increasing variety of sources for health information, people tend to look for what concerns them online due to ease of access [9]. They may prefer online consultations from doctors they have never met to make an appointment at a clinic and visiting their physician [10].

After the recent COVID-19 pandemic, most individuals obtained their information from online sources, and more online applications were introduced to the people, whether from governmental or individual work [11]. These platforms and search engines were a convenient way to communicate with doctors around the globe and to find out the causes for their symptoms [12]. Such methods have helped many patients find the answers they seek with ease and with less cost, but it comes with a higher risk of obtaining misleading information through untrustworthy platforms [13].

Many studies were conducted to see the people’s perception, and their attitude toward the use of the Internet for health information [14-15]. Such studies were also carried out in Saudi Arabia, but most have been limited to a specific target sample of the population, generally the higher educated, which limits its generalizability [11, 13, 16].

Our study aimed to assess the perception towards the use of different platforms and search engines to seek health information in Saudi Arabia.

## Materials and methods

Study design

This is a non-interventional cross‐sectional study. It was conducted in the Kingdom of Saudi Arabia from January 2021 to July 2021. All who are capable of using the Internet, social media, or both were included. 

Data collection and tools 

An online survey was made available on various social media platforms and was accessible to all citizens and residents of the Kingdom of Saudi Arabia using these platforms. 

According to the estimated population of Jeddah for 2020, a sample size required for this study was calculated to be at least 385 participants with a 95% confidence level and a margin of error of 5%; the calculations were made using the Raosoft sample size calculator [17] derived from the estimated total population based on the General Authority for Statistics in the Kingdom of Saudi Arabia for the year 2020. A total of 1363 online questionnaires were submitted. 

An Arabic online self-administered questionnaire using Google forms was made available online. The questionnaire was previously developed and validated in King Fahad Medical City (KFMC), permission to use the questionnaire was obtained from previous authors [13].

The questionnaire consisted of four sections and a total of 25 questions, the sociodemographic section (age, gender, marital status, education level, nationality, income, job, the next three sections involved closed-ended and multiple choices questions about the participant's point of view on social media and the Internet). Likert scales (three- and five-point) were used for the perceptions, attitudes, and practices questions.

Analysis

Microsoft Excel 2016 was used for data entry, and statistical analysis was performed using IBM© SPSS© Statistics version 25 (IBM© Corp., Armonk, NY, USA). Qualitative data were presented as numbers and percentages and the Chi-square test (χ2) was applied to test the relationship between variables. Quantitative data were presented as mean and standard deviation (Mean ± standard deviation, SD) and the Mann-Whitney test was applied for non-parametric variables. A p-value of less than 0.05 was considered statistically significant.

Research ethics

This study was approved by the Institutional Review Board (IRB) of the biomedical ethical committee at King Abdulaziz University Hospital (Ref: 660-20). All participants were notified about the study objectives and response confidentiality, and we took their consent. All their data remained confidential and was accessed by the research team members only. Furthermore, no names or ID numbers were taken to complete the data collection form. 

## Results

Among the 1363 participants, 56.2% were females and 43.8% were males with a mean age of 30.73 ± 12.3. The majority were living in the Western region of Saudi Arabia and are Saudi. The educational level of most of the participants was undergraduate 67.5%. The common medical conditions reported by the participants were hypertension (6.2%), respiratory diseases (5.5%), and diabetes mellitus (4.4%). See Table 1.

**Table 1:** Demographics. SD, standard deviation

Variable	No. (%)
Age (mean ± SD) years	30.73 ± 12.3
Gender	
Female	766 (56.2)
Male	597 (43.8)
Nationality	
Saudi	1237 (90.8)
Non-Saudi	126 (9.2)
Marital status	
Single	765 (56.1)
Married	562 (41.2)
Divorced	30 (2.2)
Widower	6 (0.4)
Region	
Western region	1024 (75.1)
Central region	132 (9.7)
Eastern region	117 (8.6)
Southern region	68 (5.0)
Northern region	22 (1.6)
Educational level	
Uneducated	5 (0.4)
Elementary school graduate	2 (0.1)
Middle school graduate	16 (1.2)
High school graduate	270 (19.8)
Undergraduate	920 (67.5)
Postgraduate	150 (11.0)
Monthly income	
10,000 - 15,000	211 (15.5)
15,000 - 20,000	113 (8.3)
5000 - 10,000	209 (15.3)
Less than 5000	747 (54.8)
More than 20,000	83 (6.1)
Medical conditions	
Hypertension	84 (6.2)
Respiratory diseases	75 (5.5)
Diabetes mellitus	60 (4.4)
Kidney disease	15 (1.1)
Cardiovascular diseases	13 (1.0)
Liver disease	7 (0.5)
Other	194 (14.2)

Table 2 shows that the most commonly used social media platforms were WhatsApp (91.5%), YouTube (84.6%), and Twitter (82.6%). Of the participants, 76.1% used social media to obtain medical information regarding their health and most of them (92.6%) used the Internet to obtain medical information regarding their health condition or a family member. Some 78.9% found medical information about their health condition through medical websites on the Internet, and the most common medical websites browsed were the Saudi Ministry of Health (67%) and the Food and Drug Administration (54.4%).

**Table 2:** Social media use for health-related information.

Variable	No. (%)
Type of social media used	
WhatsApp	1247 (91.5)
YouTube	1153 (84.6)
Twitter	1126 (82.6)
Snapchat	1091 (80)
Instagram	1054 (77.3)
Facebook	267 (19.6)
Skype	66 (4.8)
Have you ever used social media to obtain medical information?	
Yes	1037 (76.1)
No	326 (23.9)
Have you ever used the Internet to obtain medical information?	
Yes	1262 (92.6)
No	101 (7.4)
For those who ever used the Internet to obtain medical information regarding your health condition or a family member (No.:1262)	
Where do you find medical information about your health condition?	
Medical websites	1076 (78.9)
Physician	1045 (76.7)
Search engine	977 (71.7)
Medical articles and books	625 (45.9)
A family member or friend	581 (42.6)
Medical websites browsed:	
Saudi Ministry of Health	914 (67.1)
Food and Drug Administration (FDA)	742 (54.4)
World Health Organization (WHO)	617 (45.3)
Webteb	468 (34.3)
Mayo Clinic	441 (32.4)
Daily Medical Info	435 (31.9)
Altibbi	380 (27.9)
Medscape	274 (20.1)
Webmd	271 (19.9)
Drugs.com	185 (13.6)
What is the most important topic to you that you often browse on the Internet or follow-on social media?	
Disease symptoms	1048 (76.9)
Medication Side Effects	966 (70.9)
Diagnoses and other medical conditions	950 (69.7)
Treatment of specific disease	871 (63.9)
Patient’s experience	832 (61.0)
Health awareness	788 (57.8)
Self-care	763 (56.0)
Medication Information	684 (50.2)
Medication dosing	607 (44.5)
Child health	584 (42.8)
Herbal treatment	541 (39.7)
Physician rating	488 (35.8)
Hospital rating	340 (24.9)

The most important topics the participants often browsed on the Internet or followed on social media were disease symptoms (76.9%), medical side effects (70.9%), treatment of specific disease (69.7%), and diagnosis of medical conditions (61%).

Table 3 shows that 40.1% of the participants had a medical consultation online from a doctor, of them, 72.8% applied the treatment prescribed by the doctor, 74.8% care if the prescribed medication is approved by the FDA, and most of them (67.8%) trusted the online consultant physician. 

**Table 3:** Online medical consultation.

Variable	No. (%)
Have you ever had a medical consultation online with any doctor? (No: 1363)	
Yes	546 (40.1)
No	817 (59.9)
If you answered yes, please answer the following: (No.:546)	
Do you apply the treatment prescribed by the doctor from whom you asked for an online consultation?	
Yes	397 (72.8)
No	149 (27.2)
Do you care if the prescribed medication approved by FDA?	
Yes	407 (74.8)
No	138 (25.2)
Do you trust the physician whom you consulted online?	
Yes	370 (67.8)
No	176 (32.2)

**Figure 1:**
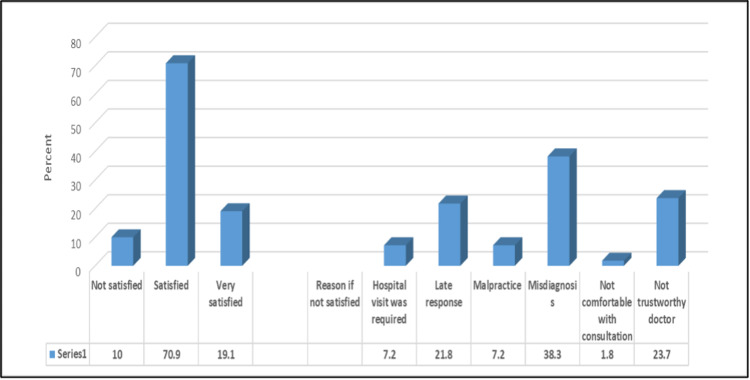
Satisfaction with online medical consultation.

Figure 1 showed that the majority (70.9%) were satisfied with the online medical consultation, and the most common reasons for dissatisfaction were misdiagnosis (38.3%), and lack of trust in the physician (23.7%). 

Table 4 shows that for the participants who seek Internet medical advice, the most common reason was that they did not want to go to the hospital (42%), of them, 78% reported that the obtained medial information helped them get a second opinion about their health condition. Some 72.5% claimed that it influenced their choice of a particular doctor, or a particular hospital and 78.7% reported that information available on social media affected how they deal with their health condition. Less than half (42.3%) reported that they tried a treatment for someone who has gone through the same experience or illness as them from social media, 46.5% reported that media influenced their decision to take certain medications, and 35.4% reported that they searched for health-related information on the Internet monthly. Most of the participants (56.4%) usually search online about their health condition before visiting their physician.

**Table 4:** Effect of social media platforms on health making decisions (No.: 1262).

Variable	No. (%)
What is your reason to seek medical advice from social media?	
You don't want to go to the hospital	530 (42.0)
I'm afraid of getting infected with any other disease while I'm at the hospital	342 (27.0)
Online consultations are free of charge	335 (26.5)
Not enough time to go the hospital	308 (24.4)
Doctors in social media give useful medical advice	282 (22.3)
Does the health information you obtained through social media help you get a second opinion about your health condition?	
Yes	984 (78.0)
No	278 (22.0)
Does social media influence your choice of a particular doctor or a particular hospital?	
Yes	915 (72.5)
No	347 (27.5)
Does the health information available on social media affect how you deal with your health condition, follow a certain diet, or practice a certain sport routine?	
Yes	992 (78.7)
No	270 (21.3)
Have you ever tried the treatment for someone who has gone through the same experience or illness as you from social media?	
Yes	533 (42.3)
No	729 (57.7)
Does social media influence your decision to take certain medications?	
Yes	586 (46.5)
No	676 (53.5)
How often (on average) do you search for health-related information on the Internet?	
Daily	57 (4.5)
Weekly	270 (21.4)
Monthly	460 (35.4)
Yearly	394 (31.2)
I rarely search	66 (6.3)
I never search	15 (1.2)
Do you research online about your health condition before visiting your physician?	
Always	343 (27.2)
Usually	424 (56.4)
Sometimes	235 (18.6)
Rarely	139 (11.0)
Never	121 (9.6)
Do you search online about your health condition after visiting your physician?	
Always	180 (14.2)
Usually	311 (24.9)
Sometimes	222 (17.5)
Rarely	220 (17.4)
Never	329 (26.0)

**Figure 2:**
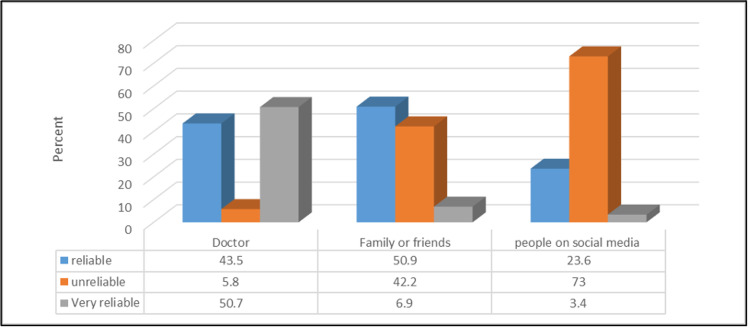
Social media credibility regarding health-related decisions (No.: 1262).

Figure 2 shows that as for the credibility of the advice given by doctors online, the participants found that family and friends (50.9%), doctors (43.5%), and people on social media (23.6%) were reliable.

Table 5 shows that for 59.6% of participants who obtained Internet medical information, the information found online was reliable, and for those who found it unreliable the most common reason was trust issues (52%). For all participants, most of the participants (90.9%) think health information on the Internet or social media contributes to raising the level of health awareness among the general population. Most of the participants (83.1%) were willing to use social media to communicate with their doctor and ask health-related questions. At the same time, only 22.2% shared their experience with certain diseases to the public on social media and 27.1% evaluated their experience with a particular doctor or hospital on social media.

**Table 5:** Reliability of online information.

Variable	No. (%)
For those who ever used the Internet to obtain medical information regarding your health condition or a family member (No.: 1262)	
In your opinion, how reliable is the information you find online?	
Very reliable	67 (5.4)
Reliable	753 (59.6)
Unreliable	442 (35.0)
If your choice is unreliable, please state the reason (No.: 442)	
Trust issues	230 (52.0)
Prefer in-person visit	67 (15.1)
Fear of adverse events	22 (5.0)
Not specified	123 (29.9)
Do you think that health information on the Internet or social media contributes to raising the level of health awareness among the general population?	
Yes	1146 (90.9)
No	116 (9.1)
For all participants (No.:1363)	
Would you like to use social media to be able to communicate with your doctor and ask health-related questions?	
Yes	1133 (83.1)
No	230 (16.9)
Have you ever shared your experience with a certain disease with the public on social media?	
Yes	302 (22.2)
No	1061 (77.8)
Have you ever evaluated your experience with a particular doctor or hospital on social media?	
Yes	369 (27.1)
No	994 (72.9)

Our results show that female participants, those of undergraduate educational level, those having cardiovascular diseases, respiratory diseases, or liver diseases had a significantly higher percentage of those who used the Internet to obtain medical information regarding their health condition or a family member (p < 0.05). In addition, there is significant relationship between gender (p = 0.042), educational level (p < 0.001), and online health seeking behavior. On the other hand, a non-significant relationship was found between the income level, marital status, age, and those who used the Internet to obtain medical information regarding their health condition or a family member (p > 0.05). 

## Discussion

This study aimed to assess the perception towards and use of different platforms and search engines to seek health information in Saudi Arabia.

Most of our participants refer to both the Internet (92.6%) and social media (76.1%) for health information. This finding is similar to another Saudi Arabian study, which found 68% of their respondents seek medical-related information through social media [18]. These figures reflect the widespread use of social media platforms due to their ease of accessibility and availability and their potential use in future social media-based public health promotion interventions [19].

The findings from this study showed that medical websites were the most used source of health information, followed by physicians. This contrasts with most other studies done in this region, which state that most of their population sample referred to doctors, rather than online sources, for health information [20-21]. This result may reflect the increasing dependence on online sources during the COVID-19 pandemic due to movement restrictions and the decreased willingness to risk exposure at the doctor’s office [22]. 

Though online sources may seem an inadequate source of health information, our study shows that most of our participants refer to reliable sources such as the Food and Drug Administration website and the Saudi Ministry of Health. This reduces the potential harm of misleading or wrong information by referring to reputable sites [23]. Another Saudi study also found that their participants referred to these sources and trusted them [24]. This finding suggests that the population is aware of what sources are most reliable and thus can refer to them for knowledge.

With regard to the types of social media used for health information, our study found WhatsApp, YouTube, and Twitter as the most popular, respectively. This is in concordance with most other studies, which also found WhatsApp to be the most popular source of information [16, 19]. In general, this poses concern as most WhatsApp contacts are family and friends that may not provide accurate medical advice which may mislead or misinform the receiver [25]. Hence social media can be used to improve or enhance patient care and knowledge; however, it can also create potential risks to patients [26]. One study found, approximately half of the participants who change their behavior in taking medication by stopping it or starting a new regimen were influenced by WhatsApp, indicating the significance of how this platform influences how people deal with their health. Only a few of these participants checked the accuracy of the advice that been given [9].

A recent study showed that WhatsApp users more than 65 years of age and those with elementary education were found to be the most vulnerable for misinformation. A possible reason could be due to the lower levels of education and awareness among these groups [27]. 

Another study found that those with older age may be more vulnerable to misinformation due to decline in cognitive function and abstract reasoning [28]. Our study also shows that YouTube is also almost as widely used as WhatsApp. These two social media platforms consistently remain the most widely used among the Saudi population as shown in other studies, some of which found that YouTube was among the most widely used social network [19]. Though like WhatsApp, some authors have criticized the use of YouTube for educational purposes because the content is not supervised [29].

Many participants have consulted a doctor online before and most were satisfied with the experience, the few that were not satisfied were mostly not convinced with the given diagnosis. Moreover, 23.7% of the population did not trust their online health care providers. This may hinder the care provided by the physicians, as adherence to management plans is highly dependent on a healthy patient-doctor relationship. The decreased level of trust may be due to a lack of adequate communication provided through online means, which may ultimately lead to patient dissatisfaction [20]. However, we found that more than half of the population considered physicians as a very reliable source of information which is similar to the findings in a study in which healthcare providers were the most trusted source among different populations [30].

Based on the results of our study, participants seem to be highly influenced by the healthcare information provided to them on social media platforms and find them reliable. It affects their decisions regarding whether they will visit a certain doctor or hospital, it affects their diet and sport routine, provides them with a second opinion and almost half of the participants claim that it plays a role in their decision to use or not use a medication prescribed by a doctor. Furthermore, the majority believed that social media contributes to raising health awareness. This again stressed the significant impact and influence social media has on the population’s healthcare decisions and thus proves to be a double-edged sword. Healthcare providers and government organizations ought to utilize social media to their advantage to provide the best access to healthcare knowledge.

Our study also found a significant difference between participants at different educational levels in the use of the Internet as a source of health information, those of higher education were found to search for health-related questions more than those who are uneducated or of lower education levels. Contrary to another study done by Alghamdi et al. which found no significance [21]. 

The findings of this study should encourage healthcare givers to be active users of social media, as well as increase the public awareness on usage of appropriate and trustworthy sources to obtain health information.

Limitations of this study include a sample size that may not be representative of the entire population, with a large number of responses arising from a specific region of the country. 

We recommend that future studies include a wider variety of social media platforms that have become more popular recently even among healthcare professionals such as TikTok; we also recommend that a larger population sample be used for better generalizability.

## Conclusions

The study reinforced that health information that is sought from the Internet and social media platforms has a great impact on the population emphasizing the need for credible information sources and how to access them. The results of this study will direct healthcare givers on the efficient usage of various social media platforms to give consultations and limit misinformation.

## References

[1] Laranjo L, Arguel A, Neves AL (2015). The influence of social networking sites on health behavior change: a systematic review and meta-analysis. J Am Med Inform Assoc.

[2] (2020). GCFGlobal.org. What is the Internet. https://edu.gcfglobal.org/en/internetbasics/what-is-the-internet/1/.

[3] Radcliffe D, Abuhmaid H (2021). How the Middle East used social media in 2020. SSRN.

[4] Bujnowska-Fedak MM (2015). Trends in the use of the Internet for health purposes in Poland. BMC Public Health.

[5] Andreassen HK, Bujnowska-Fedak MM, Chronaki CE (2007). European citizens' use of E-health services: a study of seven countries. BMC Public Health.

[6] Makowsky MJ, Jones CA, Davachi S (2021). Prevalence and predictors of health-related Internet and digital device use in a sample of South Asian adults in Edmonton, Alberta, Canada: results from a 2014 community-based survey. JMIR Public Health Surveill.

[7] Garcia ACF (2020). Internet Use for Searching Ethical Health Information in Portugal: A Cross Sectional Study. https://run.unl.pt/handle/10362/94995.

[8] Schwartz KL, Roe T, Northrup J, Meza J, Seifeldin R, Neale AV (2006). Family medicine patients' use of the Internet for health information: a MetroNet study. J Am Board Fam Med.

[9] Iftikhar R, Abaalkhail B (2017). Health-seeking influence reflected by online health-related messages received on social media: cross-sectional survey. J Med Internet Res.

[10] Ekeland AG, Bowes A, Flottorp S (2010). Effectiveness of telemedicine: a systematic review of reviews. Int J Med Inform.

[11] Mulrennan S, Colt H (2020). Medical information and social media in the time of COVID-19. Respirology.

[12] Ventola CL (2014). Mobile devices and apps for health care professionals: uses and benefits. Pharm Ther.

[13] Sumayyia MD, Al-Madaney MM, Almousawi FH (2019). Health information on social media. Perceptions, attitudes, and practices of patients and their companions. Saudi Med J.

[14] Van de Belt TH, Engelen LJ, Berben SA, Teerenstra S, Samsom M, Schoonhoven L (2013). Internet and social media for health-related information and communication in health care: preferences of the Dutch general population. J Med Internet Res.

[15] McMullan M (2006). Patients using the Internet to obtain health information: how this affects the patient-health professional relationship. Patient Educ Couns.

[16] Alzahrani A, Alanzi T (2019). Social media use by people with diabetes in Saudi Arabia: a survey about purposes, benefits and risks. Diabetes Metab Syndr Obes.

[17] (2021). Raosoft. Sample size calculator. http://www.raosoft.com/samplesize.html.

[18] Bahkali S, Alfurih S, Aldremly M, Alzayyat M, Alsurimi K, Househ M (2016). The prevalence of Internet and social media based medication information seeking behavior in Saudi Arabia. Stud Health Technol Inform.

[19] Mohammed W, Alanzi T, Alanezi F, Alhodaib H, AlShammari M (2021). Usage of social media for health awareness purposes among health educators and students in Saudi Arabia. Informatics in Medicine Unlocked.

[20] Alduraywish SA, Altamimi LA, Aldhuwayhi RA (2020). Sources of health information and their impacts on medical knowledge perception among the Saudi Arabian population: cross-sectional study. J Med Internet Res.

[21] Alghamdi E, Alqarni AS, Bakarman MM, Mukhtar AM, Bakarman MA (2019). Use of Internet health information among students in Jeddah, Saudi Arabia: a cross-sectional study. Global J Health Sci.

[22] Mehrotra A, Chernew M, Linetsky D, Hatch H, Cutler D (2020). The impact of the COVID-19 pandemic on outpatient visits: a rebound emerges. The Commonwealth Fund.

[23] Grajales FJ 3rd, Sheps S, Ho K, Novak-Lauscher H, Eysenbach G (2014). Social media: a review and tutorial of applications in medicine and health care. J Med Internet Res.

[24] Abdulsalam NM, Bakarman MA (2021). Use of social media in food safety in Saudi Arabia -- a preliminary study. AIMS Public Health.

[25] Alhaddad MS (2018). The use of social media among Saudi residents for medicines related information. Saudi Pharm J.

[26] Tonsaker T, Bartlett G, Trpkov C (2014). Health information on the Internet: gold mine or minefield?. Can Fam Phys.

[27] Bapaye JA, Bapaye HA (2021). Demographic factors influencing the impact of coronavirus-related misinformation on WhatsApp: cross-sectional questionnaire study. JMIR Public Health Surveill.

[28] Vijaykumar S, Jin Y, Rogerson D (2021). How shades of truth and age affect responses to COVID-19 (Mis) information: randomized survey experiment among WhatsApp users in UK and Brazil. Hum Social Sci Commun.

[29] Raikos A, Waidyasekara P (2014). How useful is YouTube in learning heart anatomy?. Anat Sci Educ.

[30] Hesse BW, Nelson DE, Kreps GL, Croyle RT, Arora NK, Rimer BK, Viswanath K (2005). Trust and sources of health information: the impact of the Internet and its implications for health care providers: findings from the first Health Information National Trends Survey. Arch Intern Med.

